# Mycorrhizal symbioses and Arctic shrubification

**DOI:** 10.1093/sumbio/qvaf009

**Published:** 2025-06-27

**Authors:** Kevin K Newsham, Peter Convey

**Affiliations:** NERC British Antarctic Survey, High Cross, Madingley Road, Cambridge CB3 0ET, United Kingdom; NERC British Antarctic Survey, High Cross, Madingley Road, Cambridge CB3 0ET, United Kingdom; Department of Zoology, University of Johannesburg, Auckland Park 2006, South Africa; School of Biosciences, University of Birmingham, Edgbaston, Birmingham B15 2TT, United Kingdom

**Keywords:** Arctic amplification, climate warming, ectomycorrhizas (ECM), ericoid mycorrhizas (ERM), shrub expansion, soil organic nitrogen

## Abstract

A unifying feature of the deciduous and evergreen shrubs contributing to the greening of the Arctic landmass—the process known as shrubification—is that their roots consistently form ectomycorrhizal or ericoid mycorrhizal symbioses with soil fungi. Here, we review the potential roles of these symbioses in shrubification, focusing on their effects on shrub nitrogen (N) acquisition from N-limited Arctic tundra soils. Enhanced shrub growth in response to warming or elevated atmospheric CO_2_ is attributable to several mycorrhizal-mediated processes associated with N acquisition, including increased frequencies of fungal symbionts on, in, or around shrub roots, elevated activities of extracellular enzymes secreted by mycorrhizal hyphae, or enhanced hyphal access to N at the permafrost thaw front. An analysis of the responses to experimental warming of 11 frequent ectomycorrhizal fungal genera shows no effects on their abundances but a 20% reduction in taxonomic richness in warmed soil, suggesting that elevated temperature selects for specific fungal taxa. The effects of warming on ectomycorrhizas are most pronounced in mesic soils. We conclude that ectomycorrhizal and ericoid mycorrhizal symbioses most probably influence shrubification, but that further research is needed to determine the processes by which they do so.

**Sustainability Statement**

This review addresses climate change impacts on root-fungal symbioses of Arctic shrubs, and hence focuses on the aims of UN Sustainable Development Goal 15 (Life on Land), specifically the cessation of biodiversity loss and the protection of terrestrial ecosystems and threatened species.

## Introduction

Polar amplification has caused the Arctic to warm almost four times faster than the rest of the Earth over the last five decades (Rantanen et al. 2022). Air temperatures in some locations have risen at rates of up to 1.7°C per decade, equivalent to approximately twice the average rate for the Arctic and seven times the global mean (Nordli et al. 2020). Owing to winter sea ice retreat releasing surplus oceanic heat absorbed during summer, interannual variation in warming is apparent, with winter air temperatures in the Arctic increasing at least four times faster than summer temperatures (Bintanja and van der Linden 2013), and with increases in active layer and permafrost temperatures of 0.8–1.2°C and 2.3–2.5°C per decade in summer and winter, respectively (Boike et al. 2018). Changes to the amount and form of precipitation are also evident across the region (Screen and Simmonds 2012). Owing to intensified surface evaporation in winter and enhanced atmospheric moisture inflow from lower latitudes in late summer and autumn, increases in precipitation in the Arctic of >50% are projected by the end of the twenty-first century (Bintanja and Selten 2014). Rainfall is also set to become the dominant form of Arctic precipitation (Bintanja and Andry 2017), with predicted increases in rain-season length of between 60 and >90 days across the region by the end of the twenty-first century (Landrum and Holland 2020).

Aside from glacier recession and the loss of sea ice and springtime snow cover (Miller et al. 2010, Cohen et al. 2014), one of the most profound consequences of climate change in the Arctic has been the widespread greening of the landmass recorded during recent decades (Myers-Smith et al. 2020). Observations made over the last 60–70 years indicate that a significant element of Arctic greening is associated with the spread of shrubs, notably representatives of the genera *Alnus, Betula*, and *Salix* [Note that plant nomenclature follows Plants of the World Online (https://powo.science.kew.org/) throughout], at high latitudes in Arctic regions of North America, Europe and Russia (Sturm et al. 2001a, Tape et al. 2006, Myers-Smith et al. 2011). This process, termed shrubification, arises from the increased height, cover, and biomass of shrubs, and typically those of taller shrub species, promoting their dominance over shorter-statured plants, which causes infilling between and within shrubby patches of vegetation and the northward expansion of the shrubline (Myers-Smith et al. 2011). Shrubification, which increases snowpack depth during winter by trapping snow around shrubs (Sturm et al. 2001b), has the potential to alter the Earth’s energy budget through its effect on the albedo of the Arctic landmass (Myers-Smith et al. 2015). Shrub expansion is also thought to promote the loss of carbon (C), partly as CO_2_ and CH_4_, from organic C present in permafrost (Parker et al. 2021), the mass of which in the northern permafrost zone is estimated to be 1330–1580 Pg (Schuur et al. 2015). Determining the causes of shrubification is hence central to our understanding of Arctic climate change processes relevant at the global scale (Mekonnen et al. 2021).

Despite the extensive literature generated on Arctic shrubification, the reasons for the spread of shrubs in the region have yet to be fully resolved (Myers-Smith et al. 2015). In a recent review, Mekonnen et al. (2021) identified several factors contributing to the success of shrubs in a warmer Arctic, focusing primarily on increasing summer air temperature, which correlates positively with shrub expansion (Berner et al. 2020). However, an important knowledge gap identified by Mekonnen et al. (2021) concerns the influence of soil microbes on shrubification, with the authors postulating a critical role for these organisms in the process through their potential influence on nutrient availability. This has recently been demonstrated for *Alnus* species, the root nodule-inhabiting bacterial symbionts of which fix atmospheric dinitrogen (N_2_) into soil, leading to the expansion of shrubs in nitrogen (N)-limited Arctic tundra (Schore et al. 2023). In this review, we similarly address how mycorrhizas, mutualistic symbioses that form between plant roots and soil fungi (Smith and Read 2008), might influence shrubification, primarily through their effects on N acquisition from tundra soil. First, we describe the ectomycorrhizal (ECM) and ericoid mycorrhizal (ERM) symbioses, the two most frequent mycorrhizas in Arctic tundra, and briefly outline the arbuscular mycorrhizal (AM) symbiosis. We then consider the responses of different plant forms to experimental warming, which suggest that ECM and ERM symbioses influence shrubification, and review the literature on the effects of warming and elevated atmospheric CO_2_ concentrations on these mycorrhizas in the Arctic. By doing so, we identify potential processes by which the symbioses might enhance shrub growth as the climate of the region changes. Finally, we identify knowledge gaps and propose priorities for research to clarify the role of mycorrhizal symbioses in Arctic shrubification.

## The ectomycorrhizal symbiosis

**Figure 1.**
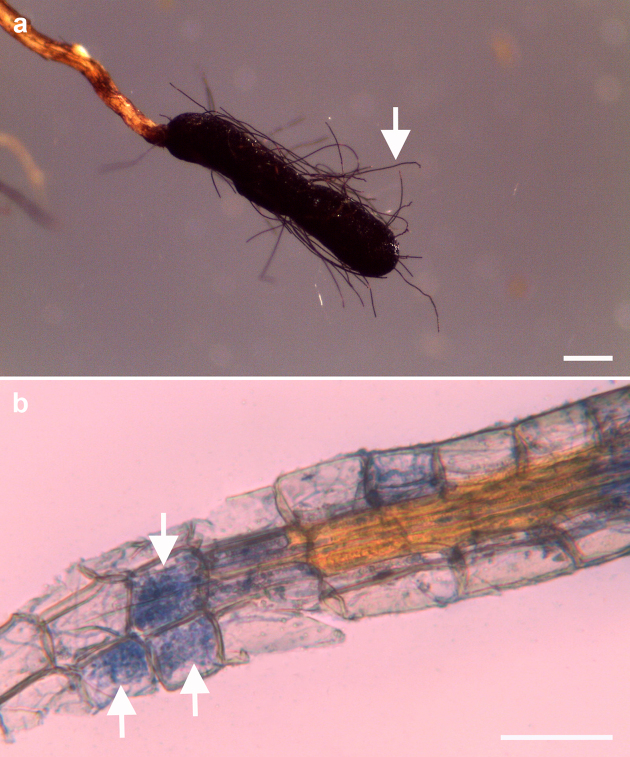
Micrographs of High Arctic plant roots showing (a) an ECM mantle with darkly pigmented emanating hyphae (arrowed) formed on a root of *Salix polaris* and (b) an ericoid mycorrhiza in the hair root cells of *Cassiope tetragona*, stained with aniline blue, showing three cells in which hyphal coils have been formed (arrowed). The scale bars in (a) and (b) are 100 µm and 50 µm, respectively.

Globally, the ECM symbiosis dominates vegetation at high latitudes and altitudes in which seasonally cold and dry climates inhibit decomposition, with boreal forest and tundra consisting predominantly of plants that form ectomycorrhizas (Read 1991, Steidinger et al. 2019). In Arctic tundra, the symbiosis routinely forms on the roots of shrubs in the families Betulaceae, Salicaceae, and Rosaceae, and particularly species of *Alnus, Betula, Salix*, and *Dryas* (Table 1). Morphologically, it is characterized by the absence of intracellular penetration of host root cells and consists of a mantle of fungal hyphae enveloping the distal root tip (Fig. 1a), a Hartig net composed of labyrinthine hyphae growing between plant epidermal and cortical cells, and fans of extraradical hyphae that grow into the soil surrounding the root (Smith and Read 2008). The ECM symbiosis plays a pivotal role in the acquisition of soil N, the principal plant growth-limiting element in Arctic tundra (Shaver and Chapin 1980, McKane et al. 2002, Schore et al. 2023). Nitrogen limitation in tundra soils is primarily caused by low temperatures and water availability, which restrict the decay of organic matter and constrain the rates of processes generating plant-assimilable inorganic N from organic forms of the element (Read 1991, Read and Perez-Moreno 2003). As a consequence, most of the huge stock of N in Arctic soils—recently estimated to weigh 21 Pg to 1 m depth (Palmtag et al. 2022)—is present in organic form, such as in chitin, proteins, peptides or amino acids, the collective concentrations of which can be several fold to orders of magnitude higher than those of inorganic N in the soils of the region (Jones and Kielland 2002, Keuper et al. 2012, Foster et al. 2016).

**Table 1.** Frequent Arctic shrub species and the mycorrhizas formed by their roots.

Shrub type	Family	Species	Mycorrhizal type	References
Deciduous	Betulaceae	*Alnus alnobetula*^‡^,*	ECM	13
		*Betula nana* ^†,‡^	ECM	2–5,7,10–12
		*Betula glandulosa* ^†,‡^	ECM	11,12
	Salicaceae	*Salix arctica* ^‡^	ECM	3,4,6–8,11,12
		*Salix herbacea × S. polaris* ^†^	ECM	2,9
		*Salix lanata* ^‡^	ECM	5,12
		*Salix lapponum* ^‡^	ECM	5,11,12
		*Salix polaris*	ECM	1,4,5,12
		*Salix reticulata*	ECM	2,4,5,7,11,12
	Ericaceae	*Vaccinium myrtillus* ^‡^	ERM	3,10,12
		*Vaccinium uliginosum*	ERM	2–4,6–8,10,12
				
Evergreen	Ericaceae	*Cassiope tetragona* ^†,‡^	ERM, ECM**	1–4,6–8,10–12
		*Empetrum nigrum*^†,‡^,^#^	ERM	4,7,10,12
		*Rhododendron tomentosum* ^†^	ERM	3,4,12
		*Rhododendron lapponicum*	ERM	2,11
		*Vaccinium vitis‐idaea* ^†,‡^	ERM	2–4,7,12
	Rosaceae	*Dryas integrifolia* ^‡^	ECM	6,8,11
		*Dryas octopetala*	ECM	1–5,7,10,12

Species marked with daggers and double daggers are those that have increased in cover, biomass, or abundance in experimentally warmed plant communities (see references 14–21) and in Arctic tundra during recent decades (see references 22–28), respectively. Abbreviations: ECM, ectomycorrhiza; ERM, ericoid mycorrhiza. References: 1, Väre et al. (1992); 2, Michelsen et al. (1996); 3, Michelsen et al. (1998); 4, Treu et al. (1995); 5, Katenin (1972); 6, Bledsoe et al. (1990); 7, Miller (1982); 8, Kohn and Stasovski (1990); 9, Clemmensen and Michelsen (2006); 10, Clemmensen et al. (2021); 11, Iversen et al. (2015); 12, Akhmetzhanova et al. (2012); 13, Black et al. (2021); 14, Chapin and Shaver (1996); 15, Hobbie and Chapin (1998); 16, Jonasson et al. (1999); 17, Graglia et al. (2001); 18, Jónsdóttir et al. (2005); 19, Jägerbrand et al. (2009); 20, Zamin et al. (2014); 21, Hollister et al. (2015); 22, Sturm et al. (2001a); 23, Tape et al. (2006); 24, Hudson and Henry (2009); 25, Molau (2010); 26, Myers-Smith et al. (2011); 27, Boulanger-Lapointe et al. (2014); 28, Vowles et al. (2017).

* Includes subspecies, notably *A. alnobetula* subsp. *alnobetula, crispa*, and *fruticosa*.

** Reported as ECM in references 7, 8 and 11.

# Includes *E. nigrum* ssp. *nigrum*.

With the exception of amino acids and short peptides, organic N cannot be directly assimilated by roots in the absence of fungal symbionts (Hill et al. 2019). Shrub roots in Arctic tundra and boreal forests hence routinely form the ECM symbiosis in order to gain access to soil organic N (Read and Perez-Moreno 2003, Newsham et al. 2009). The hyphae of ECM fungi grow beyond the nutrient depletion zones that develop around roots, secrete extracellular enzymes into soil, and transport nutrients—predominantly N but also P and other elements—to the root (Smith and Read 2008). In mature ECM symbioses in temperate ecosystems, the fans of extraradical fungal hyphae are up to 1 × 10^5^ times longer than the root system of the plant, vastly increasing the volume of soil with which the root is in contact (Read 1991). The extracellular enzymes released by ECM hyphae into soil include esterase, laccase, peroxidase, and acid proteinase, the latter of which mobilizes N from proteins (Abuzinadah and Read 1986, Read and Perez-Moreno 2003). The assimilated N is translocated through hyphae to the root, leading to the rapid uptake of amino acids from tundra soils by ECM-forming shrubs (Kielland 1994) and the depletion of amino acids in solution around shrub roots in Arctic tundra (Andresen et al. 2022). Estimates based on fractionation against the isotope ^15^N during transfer to roots indicate that 61–86% of plant N in Arctic tundra is assimilated in this way through mycorrhizal hyphal networks (Hobbie and Hobbie 2006). In addition to also absorbing inorganic N, ECM symbionts enhance P uptake by roots (Smith and Read 2008), with hyphae secreting extracellular phosphatase, enabling access to organically bound soil P (Antibus et al. 1992, Read and Perez-Moreno 2003). In return for the N and P acquired by fungal hyphae, which enhance the photosynthesis, biomass and height of plants subjected to nutrient limitation under controlled conditions (Smith and Read 2008), the plant supplies C to the fungus in the form of simple sugars fixed in photosynthesis, with approximately 8–17% of photosynthate C being transferred from aboveground tissues to ECM fungi in Arctic tundra (Hobbie and Hobbie 2006, Deslippe and Simard 2011).

A wide taxonomic range of fungi form ectomycorrhizas. With the exception of a few ascomycetes such as *Cenococcum geophilum*, the majority of these fungi are basidiomycetes, and include members of the genera *Leccinum, Cortinarius, Laccaria, Thelephora, Lactarius, Russula, Hebeloma, Tomentella, Inocybe*, and *Serendipita* (syn. *Sebacina*), which are abundant and widely distributed in Arctic soils (Geml et al. 2012, 2021, Timling et al. 2014). These genera differ in their abilities to capture nutrients from soil, primarily because of the sizes of the hyphal fans that they form in extraradical soil, the presence of hyphal aggregations termed rhizomorphs, and the hydrophobicity of their hyphae (Agerer 2006, Hobbie and Agerer 2010). Long-distance and medium-distance fringe types, such as those formed by *Leccinum* and *Cortinarius*, usually possess hydrophobic hyphae and rhizomorphs, which, in temperate ecosystems, can grow decimetres from the host plant (Agerer 2006). These taxa confer substantial benefits on host nutrition but, owing to the extensive networks of hyphae that they form, also place substantial C demands on the host plant (Lilleskov et al. 2011). Conversely, genera such as *Tomentella, Inocybe*, and *Cenococcum*, which form short- to medium-distance networks of hydrophilic hyphae that do not aggregate into rhizomorphs (Agerer 2006), place more limited C demands on their hosts but typically confer fewer benefits on host nutrient balance (Lilleskov et al. 2011).

## The ericoid mycorrhizal symbiosis

The ERM symbiosis consistently forms in the roots of predominantly evergreen Arctic shrubs in the Ericaceae, such as members of the genera *Cassiope, Empetrum*, and *Rhododendron* (Table 1). In contrast to ectomycorrhizas, the ERM symbiosis is an endomycorrhiza, in which fungal hyphae penetrate the hair root cells of ericoid plants, where hyphal coils are formed (Fig. 1b). As well as efficiently absorbing inorganic N, the ERM symbiosis plays a vital role in the absorption of N from soil organic compounds that the plant could otherwise not assimilate (Smith and Read 2008). For example, ERM symbionts secrete chitinase and other enzymes into soil around ericoid roots, including acid proteinases, which break down organic compounds such as proteins and peptides and enable amino acid uptake by the root (Leake and Read 1989, Read and Perez-Moreno 2003). The hyphae of ERM fungi also synthesize acid phosphatase and phosphodiesterase, enabling the assimilation of P from phytates and nucleic acids (Read and Stribley 1973, Smith and Read 2008). As for the ECM symbiosis, the inflow of nutrients via hyphae benefits the host plant, with experiments in which ericoid plants are inoculated with ERM fungi showing enhanced seedling N nutrition and growth under N-limited conditions (Smith and Read 2008). The diversity of the fungi forming ericoid mycorrhizas is apparently lower than that of those forming the ECM symbiosis, with pioneering studies indicating the occurrence of a single ascomycete fungus, currently known as *Pezoloma ericae* (syns. *Hymenoscyphus ericae* and *Rhizoscyphus ericae*), in the roots of ericoid plant species (Read 1991). More recent studies have confirmed *P. ericae* to be a dominant fungus forming the symbiosis, but have also shown other fungi to be present in ericoid mycorrhizas, such as the ascomycete *Meliniomyces* and the basidiomycete *Serendipita* (Hambleton and Sigler 2005, Selosse et al. 2007).

## The arbuscular mycorrhizal symbiosis

The arbuscular mycorrhizal (AM) symbiosis, an endomycorrhiza, is formed by members of the Glomeromycotina (Spatafora et al. 2016) in the root cells of forbs and graminoids in the sub-Arctic (Newsham et al. 2009). This symbiosis, which plays a pivotal role in plant acquisition of phosphorus (P) from temperate and tropical soils (Read 1991), declines sharply in abundance in Arctic tundra (Bledsoe et al. 1990, Kohn and Stasovski 1990, Barceló et al. 2023), where it is replaced by fine endophytes (Newsham et al. 2009) formed by members of the Mucoromycotina (Orchard et al. 2017). Arbuscules, the plant–fungus interface at which P is exchanged (Smith and Read 2008), usually do not form in the root cells of forbs or graminoids at high latitudes, except in closed swards of vegetation in thermal oases (Väre et al. 1992, Newsham et al. 2009, 2017). This decline in the AM symbiosis is in part attributable to the increasingly sparse vegetation found at high latitudes, which diminishes AM inoculum through reduced root-to-root contact (Smith and Read 2008), and the increased occurrence in colder environments at higher latitudes of non-mycorrhizal forb and graminoid families, i.e. families such as the Caryophyllaceae, Brassicaceae, and Cyperaceae that do not form mycorrhizal symbioses (Wang and Qiu 2006, Newsham et al. 2009).

## Responses of Arctic vegetation to warming

**Figure 2.**
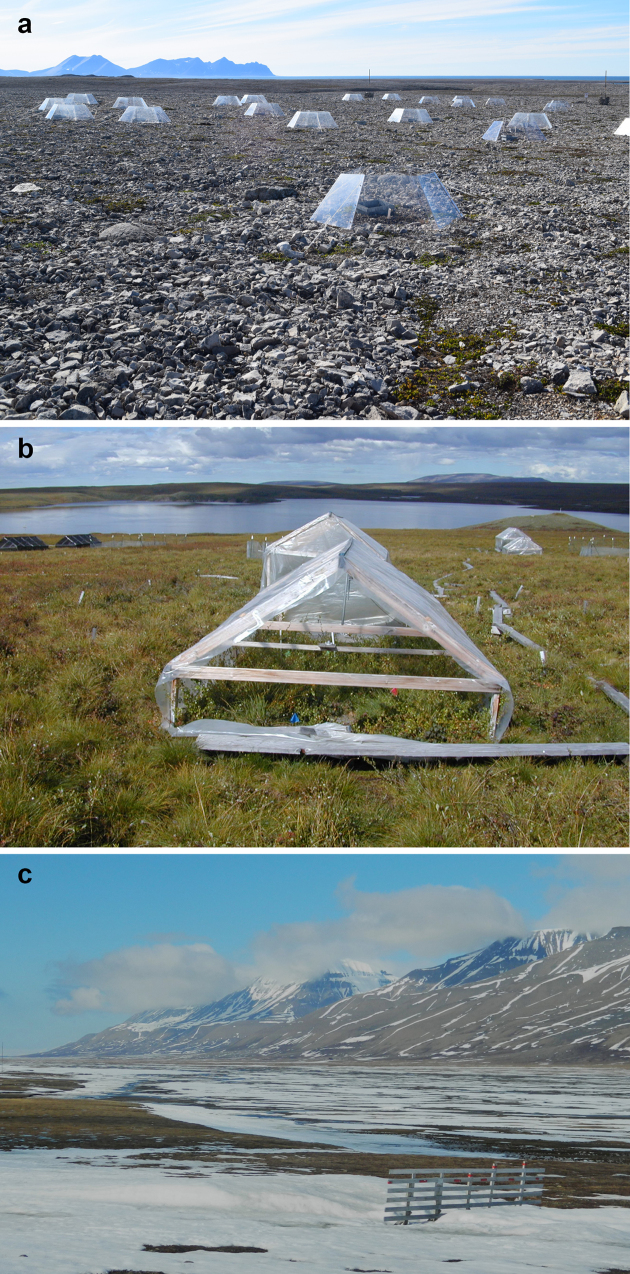
Images of (a) open top chambers deployed on Svalbard over *Salix polaris*, (b) greenhouses in Northern Alaska over moist acidic tundra dominated by *Betula nana* and *Salix pulchra* and (c) snowfences over tundra on Svalbard dominated by *S. polaris, Dryas octopetala*, and *Cassiope tetragona*. Open top chambers typically increase near-surface daily mean air temperatures by 1–3°C during summer, but also have other effects, such as altering the frequency of freeze-thaw events and soil moisture content (Marion et al. 1997, Bokhorst et al. 2011). Greenhouses similarly elevate summer near-surface soil temperatures by 1–2°C and, without vents, reduce precipitation, but have no measurable effects on soil moisture content (Clemmensen et al. 2006, Deslippe et al. 2011). Snowfences, which trap snow on their leeward sides, simulating the accumulation of snow around aboveground shrub tissues (Sturm et al. 2001b), increase minimum soil temperatures during winter by insulation, for example by 1.8°C in northern Alaska and 3.0°C on western Greenland (Morgado et al. 2016, Christiansen et al. 2018) and by at least 5°C in dry tundra on Svalbard (Moriana-Armendariz et al. 2022). Panel b is reproduced from Shaver et al. (2000), with the permission of Oxford University Press.

**Figure 3.**
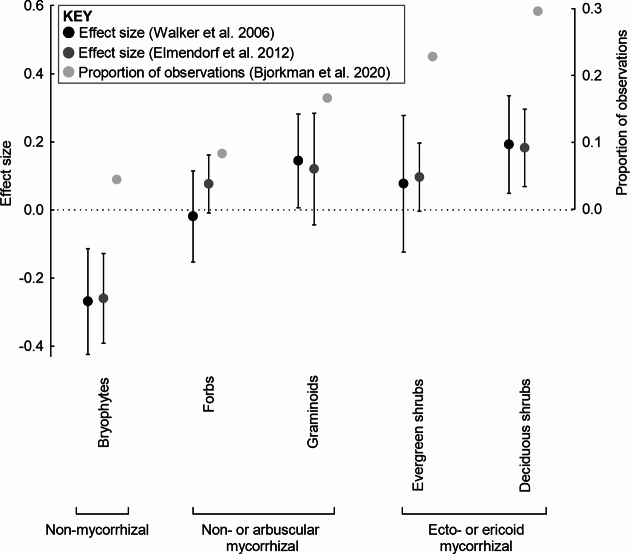
Effect sizes ± 95% confidence intervals for cover or abundance (left *y*-axis) and the proportion of observations indicating increased abundance (right *y*-axis) in response to experimental warming for bryophytes, forbs, graminoids, and evergreen and deciduous shrubs in Arctic tundra. Points for effect sizes above and below the horizontal dashed line indicate increases and decreases in cover or abundance caused by experimental warming, respectively. Note that 95% confidence intervals bars that do not cross the horizontal dotted line indicate mean values that are significantly (*P* < 0.05) different from zero, that the values for bryophytes consist predominantly of data for mosses, and that error estimates for proportional data are not available. Effect sizes, which were calculated using Hedges g or d statistics, were derived from Walker et al. (2006) and Elmendorf et al. (2012) and are based on data from experiments at 17 sites and from 61 studies, respectively. Proportional data, based on observations from 14 studies, were extracted from Bjorkman et al. (2020).

An examination of the data derived from meta-analyses of field warming experiments (Walker et al. 2006, Elmendorf et al. 2012, Bjorkman et al. 2020) suggests that plant forms routinely forming ECM and ERM symbioses in Arctic tundra usually respond more positively to warming than those that do not. These experiments have shown that warming, typically applied with open top chambers (OTCs) or greenhouses (Fig. 2a, b), consistently decreases the cover of bryophytes, and for bryophytes to be the least likely plant group to increase in abundance following experimental warming (Fig. 3). The warming-induced reductions observed in the cover and abundance of bryophytes, which consist predominantly of mosses, a non-mycorrhizal plant group (Smith and Read 2008), are attributable to shading from vascular plants (Norby et al. 2023). The cover of non-mycorrhizal or AM forbs and graminoids exhibits more positive responses to warming (Fig. 3), with the strongest responses occurring in the coldest regions of the Arctic (Elmendorf et al. 2012). The reasons for the positive effects of experimental warming on forbs and graminoids at high latitudes have yet to be fully established, but, given the sparsity of arbuscules in the roots of these plant forms in cold environments described above, it is unlikely that they are associated with benefits conferred by AM symbionts.

In contrast with the bryophytes, forbs, and graminoids, experimental warming consistently increases the cover of deciduous shrubs, with evergreen and deciduous shrubs also being the most likely plant forms to increase in abundance in warmed communities (Fig. 3). Increased shrub cover in response to elevated temperature typically occurs in mesic, as opposed to dry, tundra soils in warmer Arctic habitats (Walker et al. 2006, Elmendorf et al. 2012). Although the stature of mature *Alnus* individuals precludes tests of their responses to warming with OTCs or greenhouses, *Betula* and *Salix* species exhibit positive responses to experimental warming in the Arctic, with the growth (expressed as cover, biomass or abundance) of these ECM-forming deciduous shrubs—notably *B. nana* but also species and hybrids such as *B. glandulosa* and *S. herbacea × S. polaris—*increasing in response to elevated temperature (Table 1). Although evergreen shrubs show weaker responses to experimental warming than deciduous species, with the abundance and cover of this plant form exhibiting either non- or marginally significant positive responses to warming (Fig. 3), the growth of *Cassiope tetragona, Empetrum nigrum, Rhododendron tomentosum*, and *Vaccinium vitis-idaea* increases in experimentally warmed plant communities (Table 1). These observations are consistent with those derived from long-term surveys in the natural environment, with the cover, abundance or biomass of *B. nana, B. glandulosa, C. tetragona, E. nigrum, Dryas integrifolia*, and species of *Salix* and *Vaccinium* each having increased in Arctic tundra during recent decades (Table 1). Collectively, the enhanced growth of these ECM- and ERM-forming shrubs in experimentally warmed vegetation and the natural environment suggests that mycorrhizal symbioses may have roles in shrubification. By reviewing the literature on their responses to experimental warming and elevated concentrations of atmospheric CO_2_ in Arctic tundra, the processes by which these symbioses might do so are explored below.

## Mycorrhiza-mediated processes explaining Arctic shrubification

**Figure 4.**
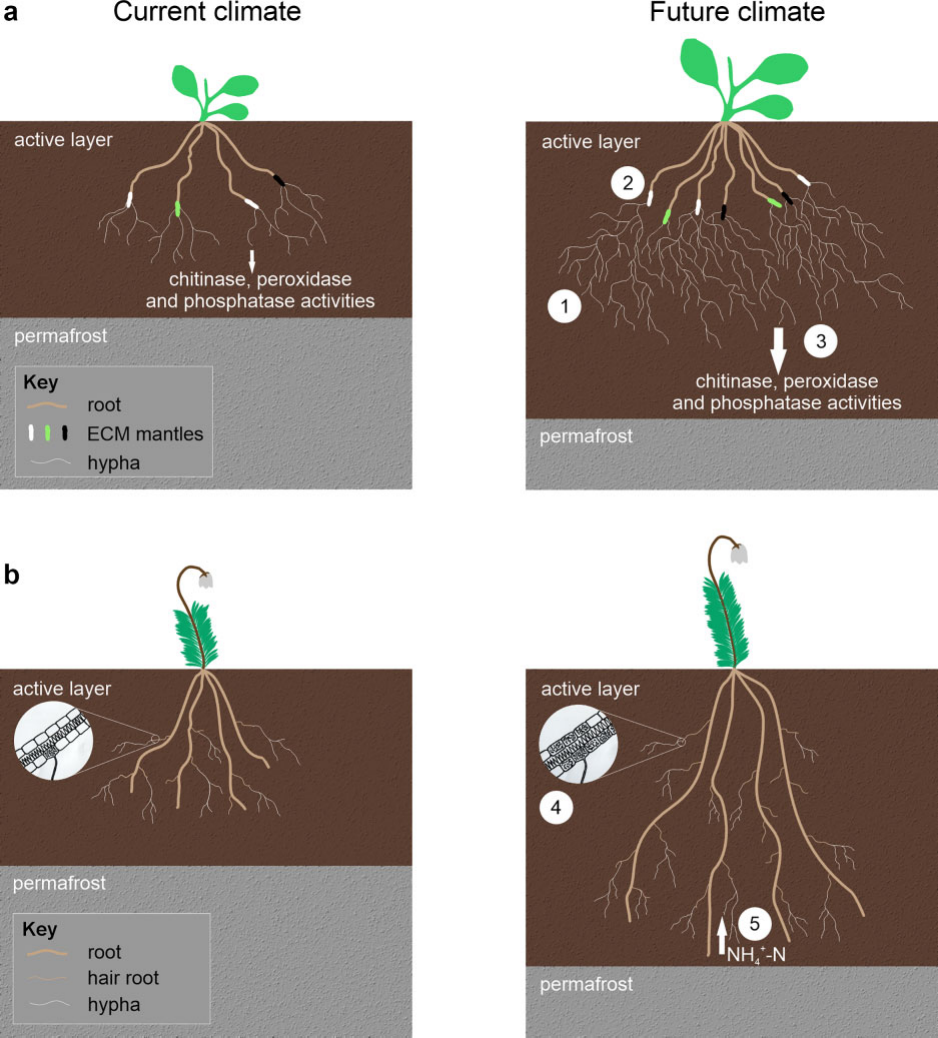
Schematic illustration of the processes by which mycorrhizal symbioses may influence shrubification in Arctic tundra in current and future climates for (a) ECM-forming and (b) ERM-forming shrubs. (1) Increased ECM soil hyphal biomass (Clemmensen et al. 2006), (2) increased ECM mantle density per volume of soil (Dunleavy and Mack 2021), (3) increased activities of extracellular enzymes secreted by ECM hyphae (Dunleavy and Mack 2021), (4) increased ERM coil frequency and biomass in ericoid hair roots (Olsrud et al. 2004, 2010) and (5) deeper ericoid root systems, with ERM hyphae transporting ammonium ions from the permafrost thaw front to roots (Hewitt et al. 2020, 2024).

Previous research in the Arctic has shown experimental warming or elevated CO_2_ to affect the biomass of ECM hyphae in tundra soil, the colonization of shrub roots by ECM- and ERM-forming fungi, the activities of extracellular enzymes secreted by mantles, the uptake of N by ERM fungal symbionts and the composition and taxonomic diversity of mycorrhizal communities. The significance of these effects as factors explaining shrubification are described below and are illustrated schematically in Fig. 4.

### ECM soil hyphal biomass

Research at Toolik Lake in Northern Alaska has shown experimental warming to increase the biomass of ECM hyphae in mesic, but not dry, tundra soils. In hydrated soil with a moisture content of 492% (per dry mass), 14 years of treatment with greenhouses increased ECM hyphal production on *B. nana* fine roots and ECM hyphal biomass (estimated by measuring concentrations of the fungal biomarker ergosterol in soil) by 39–109% (Clemmensen et al. 2006). In contrast, the same study showed warming, also applied with greenhouses for 14 years, to have no effect on ECM hyphal biomass or production on the fine roots of *B. nana* growing in a dry heathland soil at Abisko in sub-Arctic Northern Sweden with a moisture content of 110% (per dry mass). Correlative analyses showed soil ECM fungal biomass and hyphal production on *B. nana* fine roots to be positively associated, typically in the mesic soil, with aboveground plant biomass, leaf biomass, fine root biomass or fine root N concentration (Clemmensen et al. 2006). These observations are consistent with those showing warming to increase *B. nana* and *Salix pulchra* fine root N concentration and the uptake into *B. nana* tissues of ^15^N isotope injected into soil beneath shrubs (Hobbie and Chapin 1998, Clemmensen et al. 2006, Hewitt et al. 2020, 2024). Collectively, they suggest that greater ECM hyphal production and biomass in warmed soil leads to enhanced N nutrition and performance of *B. nana*, and indicate that increased hyphal biomass, allowing a greater volume of soil to be exploited for N by ECM fungi, may offer an explanation for the success of deciduous shrubs in the Arctic natural environment (Fig. 4a).

### ECM root colonization

Previous research has shown experimental warming to increase the colonization of shrub roots by ECM fungi in Arctic tundra. In a study using OTCs to warm *Salix arctica* for 5–7 years on Ellesmere Island in the High Arctic, the most parsimonious explanation for the increased efficiency of polymerase chain reaction amplification of fungal DNA from roots was greater belowground C allocation, suggesting increased ECM colonization of root tips (Fujimura et al. 2008). In contrast, no effects of 11–18 years of warming with greenhouses were found on the percentage frequencies of ECM mantles on the roots of *S. herbacea* × *S. polaris* in Northern Sweden (Clemmensen and Michelsen 2006), or on those of *B. nana* in moist acidic tundra in Northern Alaska (Deslippe et al. 2011). However, these studies did not account for the volume of soil in which mantles were formed, with further analyses showing the mean numbers of ECM-colonized root tips of *B. nana* or *S. pulchra* to increase from 0.5 mantles cm^−3^ soil in control plots to 1.3 mantles cm^−3^ soil in plots warmed with greenhouses for 28 years (Dunleavy and Mack 2021). The latter observation indicates greater abundances per volume of soil of the plant-fungal interfaces at which N and other growth-limiting nutrients are transferred to roots, corroborating the warming-induced uptake of N into shrub fine roots described above and offering a further explanation for the enhanced growth of ECM-forming shrubs in warmed soil (Fig. 4a).

### Extracellular enzyme activities

Experimental warming affects the efficiency with which ECM fungi decompose soil organic compounds. Following warming with greenhouses for 28 years in moist acidic tundra in Alaska, the activity per volume of soil of chitinase synthesized by mantles of *B. nana* and *S. pulchra* increased by ~6-fold (Dunleavy and Mack 2021). Such an increase suggests the enhanced breakdown by ECM hyphae in warmed soil of organically bound N in chitin, a primary component of fungal cell walls and invertebrate exoskeletons. Furthermore, when expressed per volume of soil, warming increased the activities of peroxidase and phosphatase synthesized by the mantles of the two shrub species by ~9- and 3-fold, respectively (Dunleavy and Mack 2021), suggesting the enhanced decomposition in soil of phenolic compounds and organically bound P, such as that present in inositol hexaphosphate, one of the major sources of soil organic P (Antibus et al. 1992). However, despite enhanced access to free amino acids being invoked as a mechanism by which ECM fungi bring about shrubification (Andresen et al. 2022), warming did not increase the activity of leucine aminopeptidase (Dunleavy and Mack 2021), an enzyme catalysing the release of N-terminal amino acids from peptides (Delange and Smith 1971). Nevertheless, the increased activities of chitinase, peroxidase and phosphatase secreted by warmed mantles suggest that enhanced shrub access to organic N and P through ECM hyphal networks may facilitate Arctic shrubification (Fig. 4a).

### ERM root colonization

Research at Abisko has shown summer warming and elevated concentrations of atmospheric CO_2_ to affect the colonization of ericoid shrub roots by fungal symbionts (Olsrud et al. 2004, 2010). Plants and soil in these studies were heated with OTCs, infra-red lamps and heating cables, maintaining a 5°C temperature difference between treated and control plots. After two growing seasons, warming more than doubled the concentration of ergosterol, a proxy for ERM colonization, in the hair roots of *Vaccinium myrtillus* and *V. vitis-idaea* relative to controls (Olsrud et al. 2004). After six growing seasons, however, microscopy showed there to be no apparent effect of warming on the abundance of hyphal coils in hair root cells (Olsrud et al. 2010). Approximately doubling atmospheric CO_2_ concentration to 730 ppm also increased the abundance of hyphal coils in the hair roots of *V. myrtillus* and *V. vitis-idaea* by 24% after six growing seasons (Olsrud et al. 2010). Ergosterol concentration correlated positively with photosynthetic rate, and, although warming and elevated CO_2_ caused 15–19% reductions in leaf N concentrations, warming increased plant cover, suggesting that N uptake per unit area of heated vegetation was not diminished (Olsrud et al. 2004). Although evidence for a direct role of ERM symbionts in the N nutrition of *Vaccinium* species exposed to warming and elevated CO_2_ concentrations was not found (Olsrud et al. 2004, 2010), the observed increases in hair roots of fungal biomass and the plant–fungus interfaces at which N is exchanged broadly suggest enhanced uptake of the element from soil into hair roots (Fig. 4b).

### ERM hyphal N uptake at the thaw front

Recent research suggests that ERM fungal symbionts transfer N from the permafrost thaw front at the base of the active layer to ericoid shrubs during summer. Nitrogen uptake by shrub species such as *C. tetragona, E. nigrum, R. tomentosum, V. uliginosum*, and *V. vitis-idaea* from ^15^N-labelled NH_4_Cl injected deep below plants correlates positively with the relative abundances of ERM-forming fungi such as *Meliniomyces bicolor* and *M. vraolstadiae* in roots, indicating that these fungi may transfer inorganic N from the permafrost thaw front to plant tissues (Hewitt et al. 2020, 2024). Challenging the view that ericaceous roots and their fungal symbionts are restricted to soil organic horizons (Read 1991), the average depth of ericoid shrub root systems doubles to ~0.4 m in soil warmed for almost three decades, with active ERM fungal hyphae being present at the thaw front (Hewitt et al. 2020, 2024). As permafrost melts across the Arctic, releasing N at the thaw front (Keuper et al. 2012, Salmon et al. 2018), these findings raise the possibility that ERM hyphae may facilitate the transport of the element from the base of the active layer to shrub roots, offering a further explanation for the spread of ericoid shrubs in a warmer Arctic (Fig. 4b).

### Mycorrhizal community composition and diversity

Approximately a dozen studies have focused on the changes caused by experimental warming to the composition and taxonomic diversity of mycorrhizal fungal communities as explanations for Arctic shrubification. However, these studies have drawn conflicting conclusions about the influence of warming on root-colonizing fungal communities. For the ERM symbiosis, OTCs deployed over tundra on Svalbard in the High Arctic exerted only a weak effect on the fungal community of *C. tetragona* roots following 5 years of treatment (Lorberau et al. 2017), whereas greenhouses affected the composition of the fungal community colonizing ericoid shrub roots in Northern Alaska after almost 30 years of treatment (Hewitt et al. 2024). For the ECM symbiosis, the abundances of mantles formed by six ECM fungal genera on the roots of *S. herbacea* × *S. polaris* did not respond to warming with greenhouses for 11 years (Clemmensen and Michelsen 2006), but OTCs deployed over *S. arctica* on Ellesmere Island increased the number of genotypes of ECM fungi present on mantles from 0.5–1.0 in untreated soil to 0.7–1.3 in warmed soil after 5–7 years of treatment (Fujimura et al. 2008). Warming with greenhouses for 18 years similarly increased the diversity of ECM fungal symbionts in the mantle community of *B. nana* in moist tundra in Alaska, with the treatment causing reductions in the frequencies of *Lactarius* species and *P. ericae*, and, remarkably, a 15-fold increase in those of the Cortinariaceae (Deslippe et al. 2011). It was proposed that high biomass, strongly proteolytic ECM, such as those formed by *Cortinarius* species, may enhance the N nutrition of *B. nana*, accounting for the responsiveness of the plant species to warming (Deslippe et al. 2011).

Summertime warming with greenhouses or OTCs also affects the composition and diversity of ECM fungal communities in Arctic soils. Automated ribosomal intergenic spacer analysis showed that, as in mantle communities, warming increased the frequency of *Cortinarius*, with 18 years of treatment causing increases in the abundance of the genus, and that of members of the Russulales and Helotiales, in mineral and organic soil horizons (Deslippe et al. 2012). More recent studies, using high-throughput DNA sequencing, have also shown strong effects of summertime warming on ECM fungal community composition in moist tundra, with reductions in the richness of *Leccinum, Tomentella, Russula, Hebeloma*, and *Inocybe* in chambered soil, but with no apparent effect on that of *Cortinarius* (Morgado et al. 2015). Strong declines in ECM community diversity have similarly been recorded in tundra soil warmed with OTCs, with reductions in the observed richness of ECM fungi from 54–138 operational taxonomic units (OTUs, i.e. groups of DNA sequences similar to each other at approximately species level) in untreated soil to 13–71 OTUs in warmed moist tundra soil after 18 years of treatment, but with no effects of warming on ECM richness in dry tundra (Morgado et al. 2015, Geml et al. 2021). Warming applied for 18 years in moist, but not dry, tundra soil also approximately halves the number of OTUs of ECM fungi with long, contact and short exploration types, and similarly halves the number of taxa with hydrophilic and hydrophobic hyphae (Morgado et al. 2015).

Snowfences, which are used to deepen snowpack and hence elevate winter soil temperatures in Arctic tundra (Fig. 2c), strongly diminish ECM fungal community richness in soil. In mesic tundra on Svalbard, snowfences reduced ECM fungal richness from 58 OTUs in control soil to 49 OTUs in treated soil after six years (Mundra et al. 2016). They similarly reduced the richness of the ECM fungal community in moist tundra soil in Northern Alaska after 18 years of treatment, with a reduction from 54 OTUs in control plots to 31 OTUs in treated plots, but had no effects in dry tundra (Geml et al. 2021). In contrast, deepened snowpack halved the richness of the ECM community in dry heath tundra, with 54 and 27 OTUs being recorded in control and treated soil, respectively, but had no effect on ECM richness in moist tundra soil (Morgado et al. 2016). Eighteen years of treatment with snowfences also reduced the richness of ECM fungi in both moist and dry tundra soil in Alaska, but only reduced the abundance of the symbionts in the former soil (Semenova et al. 2016). In dry tundra, deeper snowpack increased the abundance of ECM symbionts (Semenova et al. 2016), which may be attributable to increased water availability in treated soil during early summer (Moriana-Armendariz et al. 2022).

**Figure 5.**
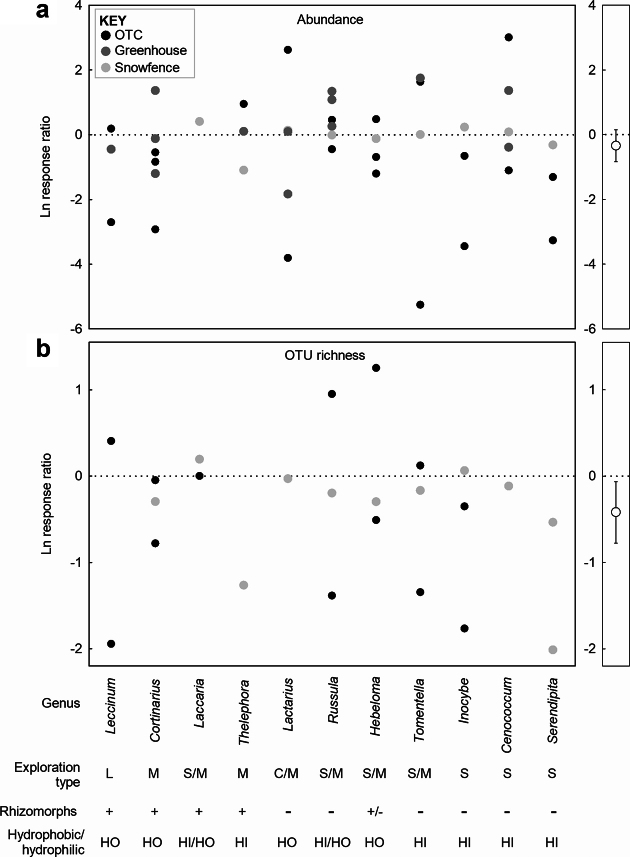
Natural logarithms of response ratios (treatment mean/control mean) for the (a) abundance and (b) OTU richness of 11 ECM-forming genera in experiments using OTCs, greenhouses or snowfences to warm plants and soil. The panels on the left show individual observations for each genus, with shading denoting different warming methods (see key in a). Points above or below the horizontal dotted lines indicate increases or decreases in abundance or OTU richness in warmed root or soil communities relative to controls, respectively. The panels on the right show the mean natural logarithms of response ratios ± 95% confidence intervals across the 11 genera. Note that 95% confidence interval bars that do not cross the horizontal dotted lines indicate mean values that are significantly (*P* < 0.05) different from zero. In (a), the response ratios derived from greenhouses were calculated from the absolute abundances of genera in mantle communities, and the ratios from OTCs and snowfences were calculated from the relative abundances of genera in roots and soil. Response ratios in (b) were calculated from OTU richness in soil. Genera are arranged with long- and medium-distance exploration types with rhizomorphs and hydrophobic hyphae to the left, and short distance exploration types lacking rhizomorphs and with hydrophilic hyphae to the right. Abbreviations: L, long distance; M, medium distance; S, short distance; C, contact; HO, hydrophobic; HI, hydrophilic. Exploration types are from Agerer (2006). See Supplementary Table S1 for the data used to draw the figure, which were derived from Clemmensen and Michelsen (2006), Deslippe et al. (2011), Deslippe et al. (2012), Morgado et al. (2015), Geml et al. (2016), Mundra et al. (2016), Semenova et al. (2016), Lorberau et al. (2017) and Dunleavy and Mack (2021). Note that the calculation of Hedge's g and similar metrics (Hedges 1981) was not possible owing to seven of the articles not reporting error data.

In an attempt to resolve these wide differences in the responses of ECM-forming fungi to warming, data from nine articles reporting the effects in the Arctic of OTCs, greenhouses and snowfences on the abundance and OTU richness of 11 frequent ECM fungal genera were analysed. A one-tailed *t*-test showed that the mean natural logarithm of response ratios (Hedges et al. 1999) for the relative or absolute abundances of the 11 genera in mantle or soil communities did not differ from zero (*t*-value −1.39, *P* = 0.173), indicating no measurable effect of warming on their abundances, and with no apparent differences between the response ratios derived from experiments using OTCs, greenhouses or snowfences (Fig. 5a). However, the same analysis showed the mean logarithm of the response ratios for the OTU richness of the ECM genera in soil to be significantly lower than zero (*t*-value −2.44, *P* = 0.023), with an average value of −0.42 across all 11 genera, and back-transformations showing a 20.2% reduction in OTU richness in warmed soil relative to controls (Fig. 5b). Although low replication precluded the analysis of data for individual genera, the logarithms of response ratios for *Serendipita* were consistently less than zero (Fig. 5a, b), suggesting that the abundance and OTU richness of the genus is diminished by warming. No clear difference emerged from the analysis between the responses to warming of ECM genera with long- or medium-distance exploration types and those with short-distance exploration types lacking rhizomorphs and with hydrophilic hyphae (Fig. 5a, b).

## Knowledge gaps and further research

The information reviewed here points to significant roles of the ECM and ERM symbioses in Arctic shrubification. However, it also exposes considerable gaps in current knowledge about the effects of climate change on mycorrhizas in the Arctic, and the processes by which the symbioses might bring about shrubification. Perhaps the widest knowledge gap concerns the response of the ECM symbiosis in Arctic tundra to elevated atmospheric CO_2_ concentrations, one of the principal factors driving global climate change (IPCC 2023). Although Arctic field experiments are challenging to install and maintain, previous studies have nevertheless successfully deployed and operated CO_2_ fumigation systems in Northern Sweden (Olsrud et al. 2004, 2010), and, given the strong effects of elevated CO_2_ concentration on the abundance and activity of the ECM symbiosis at lower latitudes (Pickles et al. 2012), there is a pressing need for further studies to assess its impacts on ectomycorrhizas in the Arctic. The influence of enhanced water availability on mycorrhizal responsiveness to warming should also be addressed by further research, particularly as rainfall is now an increasingly frequent feature of the Arctic climate (Bintanja and Andry 2017, Pedersen et al. 2022). It is evident from the literature reviewed here that water availability amplifies mycorrhizal responses to warming, with soil hyphal biomass and mycorrhizal fungal community diversity typically responding to warming in mesic, but not dry, tundra soils (Clemmensen et al. 2006, Geml et al. 2021). These observations, which are consistent with those showing shrub expansion to usually occur in warmed mesic soils (Walker et al. 2006, Elmendorf et al. 2012), further support the view that the ECM and ERM symbioses influence shrubification.

Previous studies have invoked warming-induced changes to the abundances of ECM symbionts as processes accounting for shrubification (e.g. Deslippe et al. 2011, 2012, Geml et al. 2016). However, the analyses presented here show no consensus evidence for changes to the abundances of 11 frequent ECM genera caused by warming. Nevertheless, the observation of reduced OTU richness of these genera in warmed soil, reflecting the reductions in vascular plant diversity in experimentally warmed Arctic tundra (Walker et al. 2006, Elmendorf et al. 2012, Hollister et al. 2015), suggests that elevated temperature selects for distinct ECM species (Deslippe et al. 2011), and presumably those remaining physiologically active and conferring benefits on shrubs in warmer soils. Further studies, incorporating high throughput sequencing of nucleic acids and measurements of extracellular enzyme activities (Dunleavy and Mack 2021) and fractionation against ^15^N during hyphal transfer to shrubs (Hobbie and Hobbie 2006), should identify these ECM symbionts and their influence on shrubs. Rather than applying isotopically labelled inorganic N compounds to soil (Hobbie and Chapin 1998, Hewitt et al. 2020, 2024), these studies should instead introduce ^15^N-labelled amino acids or peptides into soil beneath shrubs in order to confirm the existence of a direct link between the assimilation of soil organic N by mycorrhizal hyphae and enhanced shrub performance at elevated temperatures. Given that ^15^N tracer accumulates in roots and soil (Templer et al. 2012), and that plant belowground N concentration responds to warming (Hobbie and Chapin 1998, Clemmensen et al. 2006, Hewitt et al. 2024), these experiments should focus on the uptake of N into roots and other belowground organs, such as rhizomes.

Previous research that has determined the effects of simulated climate change on mycorrhizas in the Arctic has focused on the ECM symbiosis. Owing to this, the role of the ERM symbiosis in shrubification is presently less clear. Expanding upon previous research on *Vaccinium* species (Olsrud et al. 2004, 2010), further studies should measure the responses to warming and elevated CO_2_ of ERM fungal symbionts in the hair root cells of other ericoid shrub species that have increased in abundance in the natural environment during recent decades, notably *C. tetragona* and *E. nigrum* (Myers-Smith et al. 2011). As for the ECM symbiosis, these studies should determine the effects of warming on ERM hyphal biomass in soil and the activities of extracellular enzymes, both in the rooting zone and at the permafrost thaw front (Hewitt et al. 2020, 2024). Further research should also identify if the typically weaker responses to warming of ERM-forming shrubs than those of ECM-forming shrubs (Walker et al. 2006, Elmendorf et al. 2012) are explained by the reductions in warmed soil identified here in the abundance and OTU richness of *Serendipita*, a frequent ERM symbiont (Selosse et al. 2007).

During recent decades, species of *Alnus*, typically *A. alnobetula* and its subspecies, have spread rapidly across northern Alaska, the western Canadian Arctic and Arctic Russia (Tape et al. 2006, Myers-Smith et al. 2011). Unlike other shrubs that have increased in cover and abundance in Arctic tundra, such as *Betula* and *Salix* species, *A. alnobetula* forms tripartite symbioses with ECM fungi and root nodule-inhabiting Actinobacteria in the genus *Frankia*, the latter of which are thought to be responsible for the success of shrubs in the Low Arctic owing to their fixation of atmospheric N_2_ into soil (Schore et al. 2023). Although likely to be hampered by the tall stature of *A. alnobetula*, which attains heights of >1 m in the Low Arctic (Black et al. 2021), the benefits that it derives from its actinobacterial and ECM symbionts at ambient and elevated temperatures should be identified. Actinobacteria are typically mesophiles, with isolates of *Frankia* from *Alnus* in alpine habitats being incapable of growth at <15°C (Moiroud et al. 1984). In contrast, some ECM fungi, such as *Hebeloma crustuliniforme* isolated from *Alnus incana* subsp. *tenuifolia* in Arctic taiga, are psychrophiles that exhibit protease activity at temperatures as low as 2°C (Tibbett et al. 1999). Identifying the influence of soil temperature on the relative benefits conferred on *A. alnobetula* by its actinobacterial and ECM symbionts may hence be a productive avenue for future research.

Rising soil temperatures associated with climate change will enhance organic matter decomposition in hydrated Arctic tundra soils (Aerts 2006), with shrubification substantially influencing soil stoichiometry through its positive effects on the mineralization rates of N and P relative to C (Chen et al. 2022), and with encroachment by *Salix* spp. causing increased soil N:P ratios (Du et al. 2024). Previous research has shown that inorganic N and P applied yearly at rates of 5–10 g m^−2^ and *c*. 1–5 g m^−2^, respectively, which increase inorganic nutrient availability to at least 10 times the annual uptake requirements of tundra vegetation (Shaver and Jonasson 1999, Clemmensen and Michelsen 2006), typically counteract the effects of warming on the ECM symbiosis, with fertilization reducing ECM diversity and the activities of extracellular enzymes (Deslippe et al. 2011, Dunleavy and Mack 2021). Further studies are needed to identify the extent to which moderate inputs of inorganic nutrients, at, for example, the annual uptake requirements of shrubs, diminish the effects of warming on the ECM and ERM symbioses, with the aim of determining whether future increases in soil inorganic N and P availability and associated changes to soil stoichiometry might influence the benefits conferred by mycorrhizas on shrubs in a warming Arctic.

Mycorrhizal symbioses have profound impacts not only on the cycling of N but also on that of C (Smith and Read 2008). Mycorrhizal fungal hyphae constitute a substantial C sink, with global drawdown of C into ECM and ERM hyphal networks estimated at 9.2 billion tonnes of CO_2_ equivalents per annum (Hawkins et al. 2023), and, in forests in northern Sweden, roots and associated mycorrhizal fungi accounting for 50–70% of soil C, and ECM hyphae accounting for one third of soil microbial biomass (Högberg and Högberg 2002, Clemmensen et al. 2013). However, mycorrhizal hyphae also act as sources of CO_2_. By decomposing soil organic compounds such as chitin and cellulose (Read and Perez-Moreno 2003), the two most abundant organic polymers in the natural environment (Klemm et al. 2005, Rinaudo 2006), they respire C to the atmosphere as CO_2_, counteracting C drawdown into hyphal networks. Recent research indicates that Arctic shrub expansion promotes substantial C losses from soil through respiration, which are unlikely to be offset by increases in shrub biomass (Parker et al. 2021). In the northern permafrost zone, where the mass of soil organic C is approximately double that of the C present in the atmosphere (Schuur et al. 2015), it is evident that changes to the balance of C drawdown into, and efflux from, mycorrhizal hyphal networks in warmer soils could have globally significant consequences. The quantification of C fluxes through mycorrhizal hyphal networks under shrubs in Arctic tundra is hence a priority for future research.

## Conclusions

Given the limitations imposed on plant growth by N availability in tundra, the increased cover, biomass and height of Arctic shrubs observed in the natural environment during recent decades can be attributed, at least in part, to ECM- and ERM-mediated processes associated with enhanced soil N acquisition, including increased mantle and hyphal coil frequencies on and in shrub roots, elevated extracellular enzyme activities, or enhanced hyphal access to N at the thaw front. Nevertheless, further research is required to clarify the processes by which ECM and ERM symbioses might influence shrubification and the potential consequences of these processes for the global climate. Through identifying these processes and furthering knowledge of shrub expansion in the Arctic, this research will help to inform policies to conserve terrestrial ecosystems and to preserve biodiversity, addressing UN Sustainable Development Goal 15 (United Nations Department of Economic and Social Affairs 2024).

## Supplementary Material

qvaf009_Supplemental_File

## Data Availability

There are no new data associated with this manuscript.
